# Class II phosphatidylinositol 3-kinase α and β isoforms are required for vascular smooth muscle Rho activation, contraction and blood pressure regulation in mice

**DOI:** 10.1186/s12576-020-00745-2

**Published:** 2020-03-19

**Authors:** Shahidul Islam, Kazuaki Yoshioka, Sho Aki, Kazuhiro Ishimaru, Hiroki Yamada, Noriko Takuwa, Yoh Takuwa

**Affiliations:** 1 Department of Physiology, Kanazawa University Graduate School of Medical Sciences, 13-1 Takara-machi, Kanazawa, Ishikawa 920-8640 Japan; 2 Department of Health Science, Ishikawa Prefectural University, Kahoku, Ishikawa 929-1210 Japan

**Keywords:** Class II phosphatidylinositol 3-kinase, Vascular smooth muscle, Contraction, Rho, Blood pressure

## Abstract

Class II phosphatidylinositol 3-kinases (PI3K), PI3K-C2α and PI3K-C2β, are involved in cellular processes including endocytosis, cilia formation and autophagy. However, the role of PI3K-C2α and PI3K-C2β at the organismal level is not well understood. We found that double knockout (KO) mice with both smooth muscle-specific KO of PI3K-C2α and global PI3K-C2β KO, but not single KO mice of either PI3K-C2α or PI3K-C2β, exhibited reductions in arterial blood pressure and substantial attenuation of contractile responses of isolated aortic rings. In wild-type vascular smooth muscle cells, double knockdown of PI3K-C2α and PI3K-C2β but not single knockdown of either PI3K markedly inhibited contraction with reduced phosphorylation of 20-kDa myosin light chain and MYPT1 and Rho activation, but without inhibition of the intracellular Ca^2+^ mobilization. These data indicate that PI3K-C2α and PI3K-C2β play the redundant but essential role for vascular smooth muscle contraction and blood pressure regulation mainly through their involvement in Rho activation.

## Introduction

Phosphatidylinositol 3-kinases (PI3K) are lipid kinases that catalyze the phosphorylation of phosphoinositides at the D3 position of their inositol ring and, thereby, control a diverse array of cellular processes including vesicular trafficking, cell migration, cell proliferation and cell metabolism [1]. Mammals possess eight PI3K isoforms, which are divided into three classes (class I, II and III), based on their structural homology, substrate specificity and mode of regulation [2]. Class I PI3K are activated downstream of various receptor tyrosine kinases and G protein-coupled receptors to mainly generate phosphatidylinositol-3,4,5-trisphosphates and to mediate cell proliferation, survival, and migration. Through these effects, class I PI3K are involved in cancer and allergic diseases. The unique member of class III, Vps34, is responsible for a significant fraction of phosphatidylinositol-3-monophosphate in autophagosomes and endosomes, in which it controls the formation of phagophores as well as docking and fusion of endosomes. Class II PI3K, which comprises three isoforms PI3K-C2α (C2α), PI3K-C2β (C2β) and PI3K-C2γ, mainly produce phosphatidylinositol-3,4-bisphosphate (PI(3,4)P_2_) and are involved in endocytosis, autophagy and cilia formation at the cellular level [3–8]. C2α and C2β are ubiquitously expressed widely in various organs and tissues whereas PI3K-C2γ expression is restricted mainly in liver, breast, testis and prostate [9]. C2β is highly homologous in its amino acid sequence to C2α and exhibits similar activities including cell migration and growth to those of C2α. However, the physiological function of class II PI3K is still poorly understood compared with class I and class III PI3K.

We previously showed that C2α, which is clearly less sensitive to the PI3K inhibitors wortmannin and LY294002 [10, 11], was required for membrane depolarization- and receptor agonist-induced contraction of isolated blood vessel strips and vascular smooth muscle cells [12–14]. Moreover, our study suggested that C2α was involved in Rho activation and Rho kinase-dependent phosphorylation of the myosin-targeting subunit MYPT1 of myosin light chain phosphatase (MLCP) and, thereby, an increase in phosphorylation of 20 kDa regulatory myosin light chain (MLC_20_). We further demonstrated that in vascular smooth muscle of spontaneously hypertensive rats, enhanced activation of C2α contributed to increased Rho activity, elevated vascular tone and hypertension [15]. These observations suggested that C2α is an important regulatory molecule for Rho activation and contraction in vascular smooth muscle.

In order to better understand the role of C2α at the organismal level, we generated C2α-knockout (KO) mice and analyzed their phenotype. Global C2α-homozygous KO mice were embryonic lethal owing to severe defects of angiogenesis [3]. Endothelial-specific genetic deletion of C2α recapitulated this phenotype. In contrast to C2α, global C2β-homozygous KO mice were apparently normal [16]. We, therefore, generated smooth muscle-specific C2α-deleted mice with or without global C2β KO to study the role of C2α and C2β in vascular smooth muscle contraction. Our data show that single KO of either C2α or C2β did not affect vascular smooth muscle contraction or blood pressure, but that double KO of C2α and C2β resulted in markedly attenuated vascular smooth muscle contraction with impaired Rho activation and decreased blood pressure compared with control mice. These observations indicate that C2α and C2β have the essential compensatory role for vascular smooth muscle contraction and blood pressure regulation in mice.

## Methods

### Mice

Mice were housed in a temperature-controlled (24 °C) facility room under a 12-h/12-h light–dark cycle with free access to regular chow and water under specific pathogen-free conditions in the animal facility of the University of Kanazawa. All mouse experiments were performed following the “fundamental guidelines for proper conduct of animal experiment and related activities in academic research institutions” under the jurisdiction of the Ministry of Education, Culture, Sports, Science, and Technology of Japan and were approved by the Committee on Animal Experimentation of Kanazawa University. PI3K-C2α-floxed (C2α^flox/flox^) mice, PI3K-C2β-null (C2β^−/−^) mice, and Rosa26-CAG-loxP-stop-loxP-tdTomato (R26-tdTomato) reporter mice were described previously [3, 17]. To generate smooth muscle-specific conditional C2α-KO mice, C2α^flox/flox^ mice were crossed with SM22α-Cre transgenic mice (# 004746, Jackson Lab.). To evaluate Cre-mediated recombination efficiency, SM22α-Cre; R26-tdTomato mice were generated. Mice were euthanized using intraperitoneal injections of overdoses of three combined anesthetics, medetomidine (ZENOAQ, Fukushima, Japan), midazolam (Astellas, Tokyo, Japan) and butorphanol (Meiji Seika Pharma, Tokyo, Japan) according to the acceptable euthanasia guidelines. Mice were genotyped by PCR analysis of genomic DNA prepared from tail biopsies.

### Immunofluorescent staining of aortic sections

The perfusion-fixed aortae were embedded in Tissue-Tek OCT compound (Sakura Finetek, Tokyo, Japan), and the prepared sections were blocked with phosphate buffer saline containing 0.1% Triton X-100, 0.1% Tween 20, 1% bovine serum albumin, and 5% non-immune goat serum for 1 h at room temperature. The cryosections of aorta were subjected to immunofluorescent staining using the following primary antibodies: mouse monoclonal anti-αSMA (#A5228, Sigma-Aldrich, St. Louis, MO, USA), mouse monoclonal anti-SM22α (#ab10135; Abcam, Cambridge, UK), rabbit polyclonal anti-smooth muscle myosin heavy chain 11 (#ab53219; Abcam), rabbit polyclonal anti-C2α (#AP11855B, Abgent, San Diego, CA, USA), rabbit polyclonal anti-C2β (#sc-134766, Santa Cruz Biotechnology, Dallas, TX, USA). The sections were treated with appropriate Alexa-Fluor conjugated secondary antibodies (Molecular Probes) and, then, mounted with 4′,6-diamidino-2-phenylindole (DAPI) for nuclear staining.

### Measurement of blood pressure

Male mice aged 10 to 12 weeks were used. Mice were trained by repeated blood pressure measurements in several trials until the basal condition was stabilized and the consistent and stable reading was monitored. The systolic, diastolic and mean blood pressure with the heart rate of conscious mice was recorded using a tail-cuff system (Softron, Tokyo, Japan) as reported previously [15, 18].

### Measurements of isometric tension of aortic rings

The aorta was isolated from control and DKO mice aged 10 to 12 weeks, cleaned free of adhering connective tissues and cut into rings of 3–4 mm width in ice-cold modified Krebs–Henseleit buffer as previously described [12, 14]. Aortic rings were placed in contraction chambers at 37 °C under the aeration with 95% O_2_ and 5% CO_2_, and isometric tension was determined with a transducer (#UFERUM-203, Kishimoto Medical Instruments, Kyoto, Japan) as described previously [12, 14]. Aortic rings were stimulated with various doses of KCl and noradrenaline (NA) (Cayman Chemical) in cumulative manners. When indicated, aortic rings were pretreated with the Rho kinase inhibitor Y-27632 (10 µM) (FUJIFILM Wako Pure Chemical, Osaka, Japan). After tension measurements, aortic rings were fixed in 4% formalin overnight at 4 °C and embedded in paraffin, followed by cross-sectioning and Azan staining for the determination of the medial smooth muscle area. Tensions were corrected by a cross-sectional medial smooth muscle area in each aortic ring.

### Plasmids

The enhanced green fluorescent protein (GFP)-tagged human PI3K-C2α (GFP-C2α) expression vector was previously described [3]. Human PI3K-C2β cDNA was obtained from K. Kitatani (Setsunan University) [19]. To generate mCherry-tagged PI3K-C2α (mCherry-C2α) and GFP- and mCherry-tagged PI3K-C2β (GFP-C2β and mCherry-C2β), human PI3K-C2α and PI3K-C2β cDNA fragments were amplified by PCR using Prime STAR HS DNA Polymerase (Takara, Shiga, Japan) and sub-cloned into pmCherry-C1 (Takara) and pAcGFP1-N vectors (Clontech, Mountain View, CA, USA), using the In-Fusion HD Cloning kit (Clontech). The plasmid vectors of RhoA-FRET sensor, pTriEx-RhoA-wt_mScarlet-i_SGFP2 (#85071, Addgene), and BFP-Rab5 (#49147; Addgene) were obtained from Dorus Gadella and Gia Voeltz, respectively, through Addgene.

### Aortic smooth muscle cells

Mouse aortic smooth muscle cells (MASM) were isolated from 4-week-old mouse aortae by an enzyme-dispersion method. Briefly, aortae were dissected under sterile conditions and incubated at 37 °C in 0.1% collagenase (Type II) (#LS004202, Worthington Biochemical, Lakewood, NJ, USA), 0.75 unit/mL elastase (Type III) (#LS002279, Worthington Biochemical) and 0.1% trypsin inhibitor (#LS003570, Worthington Biochemical) for 30 min, followed by further incubation of the mixtures for 60 min after separating the adventitia from aortae. Dispersed single cells were separated from undigested tissues by filtration through Cell Strainers (100 μm) (#352360, BD Falcon) and collected by centrifugation at 500*g* for 5 min. Cells thus obtained were plated onto laminin (20 μg/mL in PBS) (FUJIFILM Wako Pure Chemical Corp. Cat no. 120-05751)-coated glass bottom dishes (MatTek, Bratislava, Slovakia) with the growth medium SmGM-2, which contains 5% fetal bovine serum (FBS) and growth factor supplements (#CC-3182, Lonza, Walkersville MD, USA). Human aortic smooth muscle cells (HASM), which were purchased from Lonza (#CC-2571, Lonza), were also plated onto laminin-coated dishes and slides with SmGM-2. After cells were cultured for 3 to 4 days, cells were transfected with siRNAs and Lipofectamine RNAiMAX (Invitrogen/Thermo Fisher Scientific) in Opti-MEM (Invitrogen/Thermo Fisher Scientific) by incubating cells with siRNA was 4 to 6 h and cultured in SmGM2 for 48 h. The concentrations of siRNAs were 50 nM for the transfection of a single siRNA (single transfection) and 25 nM each for the transfection of two siRNAs (double transfection). Our preliminary studies confirmed that 25 and 50 nM of siRNAs in the single transfection and 25 nM of siRNAs in the double transfection gave the similar extents of inhibition of protein expression of our interest. The targeted sequences of siRNA were: 5′-AAG GUU GGC ACU UAC AAG AAU-3′ for human PI3K-C2α and 5′-AAG CCG GAA GCU UCU GGG UUU-3′ for human PI3K-C2β). The control siRNA sequence was 5′-AAU UCU CCG AACGUG UCA CGU-3′. We transfected cells with GFP- and mCherry-tagged PI3K expression vectors using Lipofectamine (Invitrogen/Thermo Fisher Scientific) in Opti-MEM, followed by cultures in the growth medium for 3–4 days. Cells were serum- and growth factor-starved for 1 to 4 h in DMEM supplemented with 0.1% fatty acid free BSA (Sigma-Aldrich) for contraction assay, Western blot analyses of phosphorylation of MLC_20_ and MYPT1, and Rho imaging analyses.

### Determinations of contraction and the intracellular free Ca^2+^ concentration ([Ca^2+^]_i_) of vascular smooth muscle cells

Cells were loaded with the fluorescent Ca^2+^ indicator fluo-8 acetoxymethylester (AM) (2.5 μM) (Molecular Probes) in Hank’s Balanced Salt Solution (HBSS) for 30 min in the dark at 37 °C, followed by 1 h starvation with serum-free, phenol red-free FluoroBrite DMEM (Gibco, Thermo Fisher Scientific). After cells were washed with FluoroBrite DMEM two times, cells were imaged in FluoroBrite DMEM at 37 °C using a confocal microscope (inverted IX70 microscope; Olympus Corp., Tokyo, Japan) equipped with a confocal disk-scanning unit (CSU10, Yokogawa, Tokyo, Japan) as described previously [13]. The acquisition and process were controlled by iQ software (Andor, Belfast, UK). For evaluations of cell contraction, a stimulus-induced reduction in the planar cell surface area, which was sharply visualized by Fluo-8 fluorescence, was determined and expressed as a stimulus-induced change (∆*A*) of the planar cell area over the planar cell area before stimulation (*A*_0_) [12, 13]. A stimulus-induced change in the [Ca^2+^]_i_ was determined using the fluorescent Ca^2+^ indicator Fluo-8 as described previously [13]. Changes in Fluo-8 fluorescence were monitored every 10 s with excitation at 488 nm light and emission fluorescence at 510 nm. The ratio of ionomycin-stimulated fluorescence intensity over basal intensity was calculated.

### Fluorescence resonance energy transfer (FRET) imaging for Rho activation

For FRET imaging analysis, HASM were transfected with the RhoA-FRET sensor probe [3, 17] using an Amaxa Nucleofector system (Lonza) and plated onto laminin-coated, glass-bottomed culture dishes. For the measurements of RhoA-FRET signals, the ratio imaging was carried out on the Dragonfly confocal system (Andor) equipped with the Andor’s spinning-disk unit and EMCCD camera (iXon DU888) based on an inverted Nikon Eclipse Ti2 microscope (Nikon Instrument, Tokyo Japan). Cells were stimulated with either ionomycin (IMC) (0.3 µM) or endothelin-1 (ET-1) (1 µM), after 2 min observations as baseline signals. Pseudo-color ratio images were generated from images from GFP and FRET channels using Andor iQ software. RhoA-FRET signal intensity within ten subcellular regions per cell at 3 min after the addition of agonists was quantified. The ratio of ionomycin- and endothelin-stimulated fluorescence intensity/basal intensity was expressed. In some experiments, the co-localization of RhoA-FRET signals and blue fluorescent protein–Rab5 signals was evaluated.

### Immunofluorescence staining of cells

HASM were cultured in SmGM2 at 37 °C under 5% CO_2_. Cells of the passage number between 5 and 6 were used for experiments. Cells were rinsed with Dulbecco’s phosphate-buffered saline (PBS) once and fixed with 4% paraformaldehyde in 0.1 M phosphate buffer (pH 7.4) for 10 min at room temperature, followed by permeabilization with 0.3% TritonX-100 in PBS for 15 min or with 90% chilled methanol for 5 min. After blocking in 5% normal goat serum (FUJIFILM Wako Pure Chemical Corp.) and 0.3% TritonX-100 in PBS, cells were incubated with rabbit polyclonal anti-clathrin heavy chain (1:400) (#ab21679, Abcam) overnight at 4 °C. After washing, cells were incubated with an appropriate Alexa-Fluor-conjugated secondary antibody (Molecular Probes) for 1 h at room temperature.

### Immunoblot analyses

Cells were quickly washed with PBS 48–72 h after siRNA transfection and scraped into either 2× Laemmli’s SDS sample buffer or the RIPA lysis buffer on ice. For determination of MLC_20_ and MYPT1 phosphorylation, cells were quickly rinsed and quenched by adding an ice-cold stop buffer containing 10% trichloroacetic acid, 150 mM NaCl, 2 mM DTT and 4 mM EGTA, followed by scraping into 2× Laemmli’s SDS sample buffer. The pH of cell lysates was neutralized by adding a concentrated Tris solution. Cell lysates were then boiled for 5 min and centrifuged for 5 min at 15,000 rpm. The resultant supernatants were separated on 8 to 15% SDS-PAGE, followed by electro-transfer onto polyvinylidene difluoride membranes (Immobilon-P, Millipore-Merck, Nottingham, UK) using the Trans-Blot Turbo blotting system (Bio-Rad, Hercules, CA, USA). After blocking, membranes were incubated with primary antibodies at 4 °C overnight. The antibodies used were: rabbit monoclonal anti-PI3K-C2α (1:1000) (#12402; CST), mouse monoclonal anti-PI3K-C2β (1:500) (#611342, BD Biosciences, San Diego, CA USA), rabbit polyclonal anti-Mhc11 (1:1000) (#ab53219; Abcam), monoclonal anti-20 kDa myosin light chain (MLC_20_) (1:1000) (#M4401, Sigma-Aldrich), rabbit polyclonal anti-phospho-MLC_20_ (Ser^19^) (1:500) (#3671, CST), rabbit polyclonal anti-phospho-MLC_20_ (Thr^18^/Ser^19^) (1:500) (#3674, CST), mouse monoclonal anti-myosin light chain kinase (MLCK) (1:1000) (#M7905, Sigma-Aldrich), mouse monoclonal anti-MYPT1(1:1000) (#612165, BD Biosciences), rabbit polyclonal anti-phosphorylated MYPT1 (Thr^853^) (1:1000) (#4563, CST), mouse monoclonal anti-smooth muscle-specific α-actin (αSMA) (1:1000) (#A5228, Sigma-Aldrich), mouse monoclonal anti-glyceraldehyde 3-phosphate dehydrogenase (GAPDH) (1:1000) (#016-25523, FUJIFILM-Wako Pure Chemical), and mouse monoclonal anti-β-actin (1:1000) (#010-27841, FUJIFILM-Wako Pure Chemical). Membranes were then incubated with alkaline phosphatase-conjugated secondary antibodies, anti-rabbit immunoglobulin (Ig) G antibody (1:1000) (#7054, CST) and anti-mouse IgG antibody (1:1000) (#7056, CST) for 1 h, and the protein bands were visualized by color reaction using nitro-blue tetrazolium/5-bromo-4-chloro-3′-indolylphosphate p-toluidine (FUJIFILM Wako Pure Chemical) system. Protein band intensities were determined using Image Studio lite software (LI-COR Biosciences, Lincoln, NB, USA), normalized for amounts of the internal control GAPDH or β-actin and expressed as percentages of values in control cells.

### Statistical analysis

Statistical analysis was performed with Graphpad Prism software version 8. Data are presented as means ± standard error of mean (SEM). Analysis between two groups was done with two-tailed unpaired Student’s *t*-test. For comparison between multiple groups, one or two ways ANOVA followed by Bonferroni post hoc test was used unless stated otherwise. *p* value < 0.05 was considered to be statistically significant.

## Results

### Decreased blood pressure in C2α- and C2β-double knockout mice

**Fig. 1:**
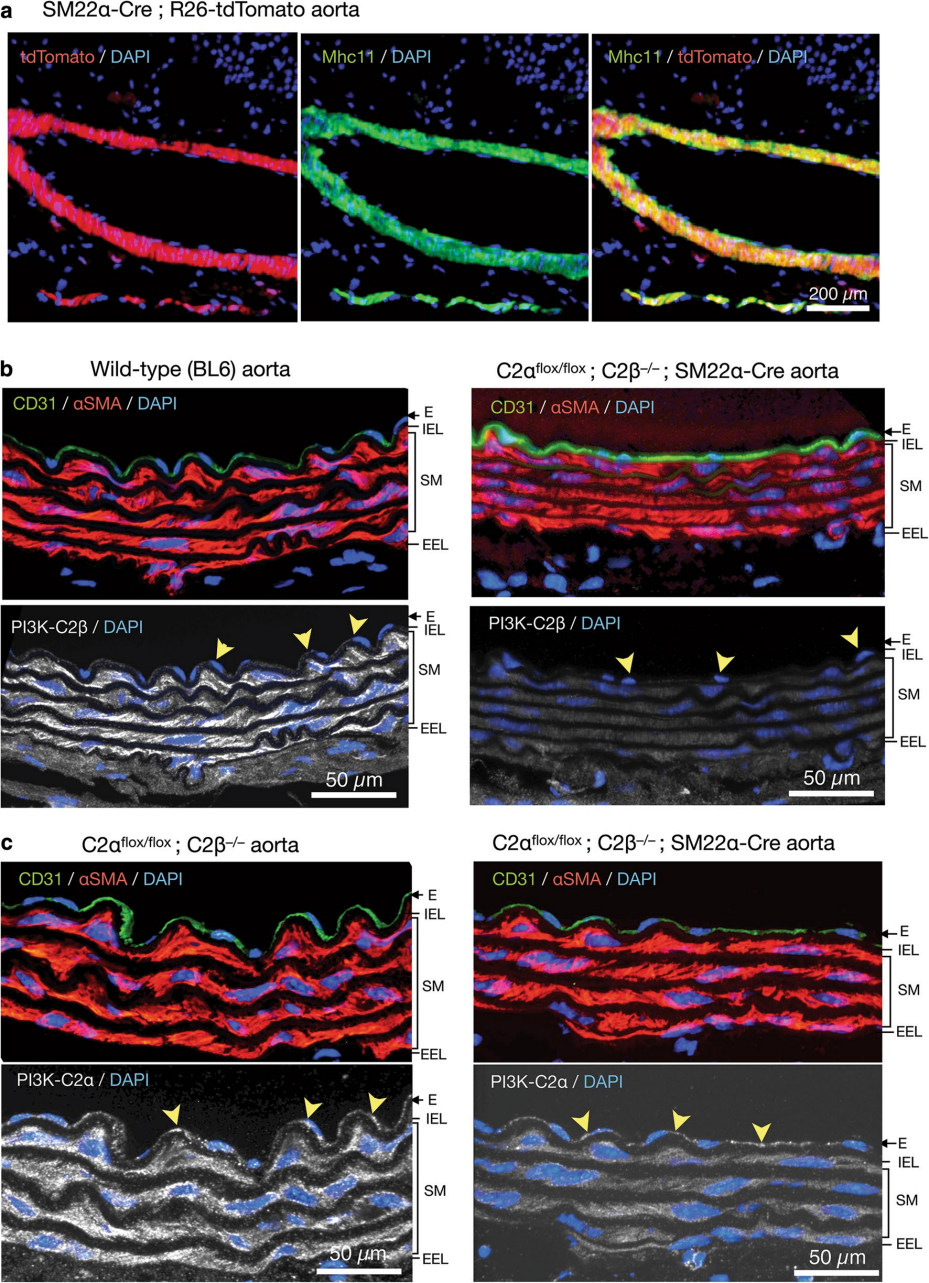
Deletion of C2α and C2β in the aortic smooth muscle layer of knockout mice. **a** The expression of tdTomato and immunofluorescence staining of myosin heavy chain 11 (Mhc11) in the medial smooth muscle layer of the aorta in SM22α-Cre; R26-tdTomato reporter mice. Mhc11-positive cells express tdTomato protein. **b** Deletion of C2β in the medial smooth muscle layer and the endothelium of the aorta in C2α^flox/flox^ C2β^−/−^; SM22α-Cre mouse. The sections of the aortic wall were immunostained using anti-C2β, anti-CD31 and anti-αSMA antibodies. The medial smooth muscle layer (SM) and the endothelium (E) were completely devoid of C2β expression in C2α^flox/flox^ C2β^−/−^; SM22α-Cre mouse (lower right), differently from wild type (BL6) mouse (lower left). *IEL* internal elastic lamina, *EEL* external elastic lamina. **c** Marked reduction of C2α expression in the SM but not the E of the aorta in C2α^flox/flox^ C2β^−/−^; SM22α-Cre mouse. The sections of the aortic wall were immunostained using anti-C2α, anti-CD31 and anti-αSMA antibodies. In **b** and **c**, yellow arrowheads indicate endothelial deletion of C2β in C2α^flox/flox^ C2β^−/−^ mouse but not of C2α in C2α^flox/flox^ C2β^−/−^; SM22α-Cre mouse, respectively. In **a**–**c**, nuclei were stained with DAPI

**Fig. 2:**
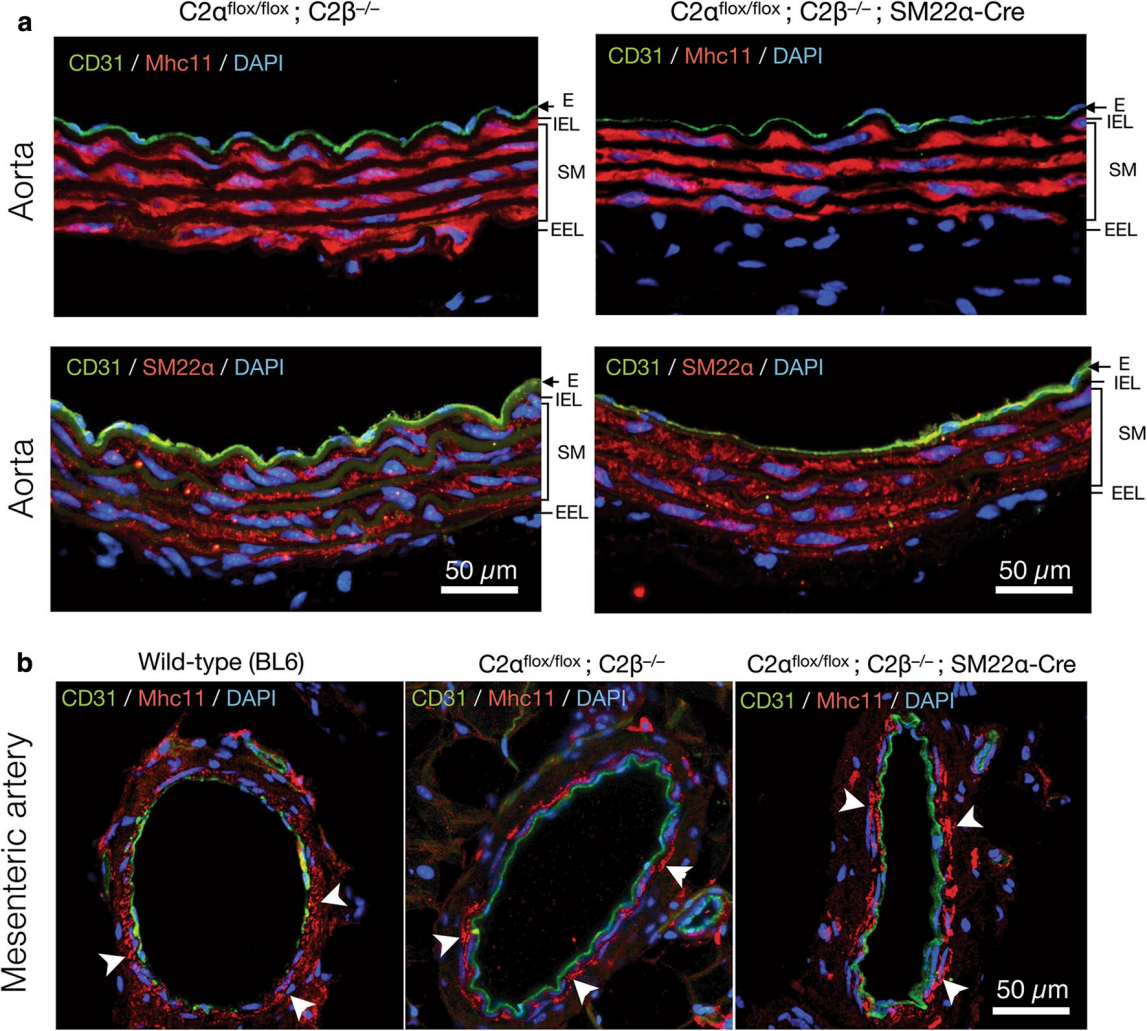
Expression of the smooth muscle-specific proteins in the aorta and mesenteric artery of control and knockout mice. **a** Immunofluorescence staining of the smooth muscle-specific proteins Mhc11 and SM22α in the aorta of control (C2α^flox/flox^; C2β^−/−^) and DKO (C2α^flox/flox^; C2β^−/−^; SM22α-Cre) mice. The SM layer was similarly stained by anti-Mhc11 and anti-SM22α antibodies. **b** Immunofluorescent staining of Mhc11 in the mesenteric artery of wild-type (BL6) (left), C2α^flox/flox^ C2β^−/−^ (middle), and C2α^flox/flox^ C2β^−/−^; SM22α-Cre mice (right). White arrowheads indicate similar expression of Mhc11 in the SM. In **a** and **b**, nuclei were stained with DAPI

To study the effects of genetic deletion of C2α and C2β on blood pressure and vascular smooth muscle contraction, we employed smooth muscle-specific C2α knockout (C2αKO) mice, global C2β-knockout (C2β^−/−^ (C2βKO)) mice, and double knockout (DKO) mice with smooth muscle-specific C2αKO and global C2βKO, which we described previously [3, 16]. Smooth muscle-specific C2αKO mice were generated by mating C2α^flox/flox^ and SM22α-Cre transgenic mice. We confirmed that SM22α promoter-driven Cre expression efficiently deleted the floxed sequence in the R26-tdTomato reporter construct, resulting in the expression of tdTomato protein in aortic smooth muscle layer which also expressed smooth muscle-specific myosin heavy chain isoform Mhc11, but not in the adventitial layer (Fig. 1a). The immunostaining of the aortic sections showed that C2β was expressed abundantly in the smooth muscle layer and slightly in the endothelium of wild-type mice but not of C2βKO mice (Fig. 1b). In control C2α-floxed (C2α^flox/flox^) and C2βKO mice, C2α was expressed in both the endothelium and smooth muscle layer of the aorta, whereas C2α expression was deficient in the smooth muscle layer but not the endothelium of smooth muscle-specific C2αKO mice (Fig. 1c). We performed morphological studies of the aorta and mesenteric artery in DKO and control mice using the immunostaining of the smooth muscle-specific proteins Mhc11 and SM22α and the endothelial marker CD31 (Fig. 2a, b). The medial smooth muscle and endothelial layers were similar in DKO and control mice, suggesting that the structure of the vasculature was not compromised in DKO mice.

**Fig. 3:**
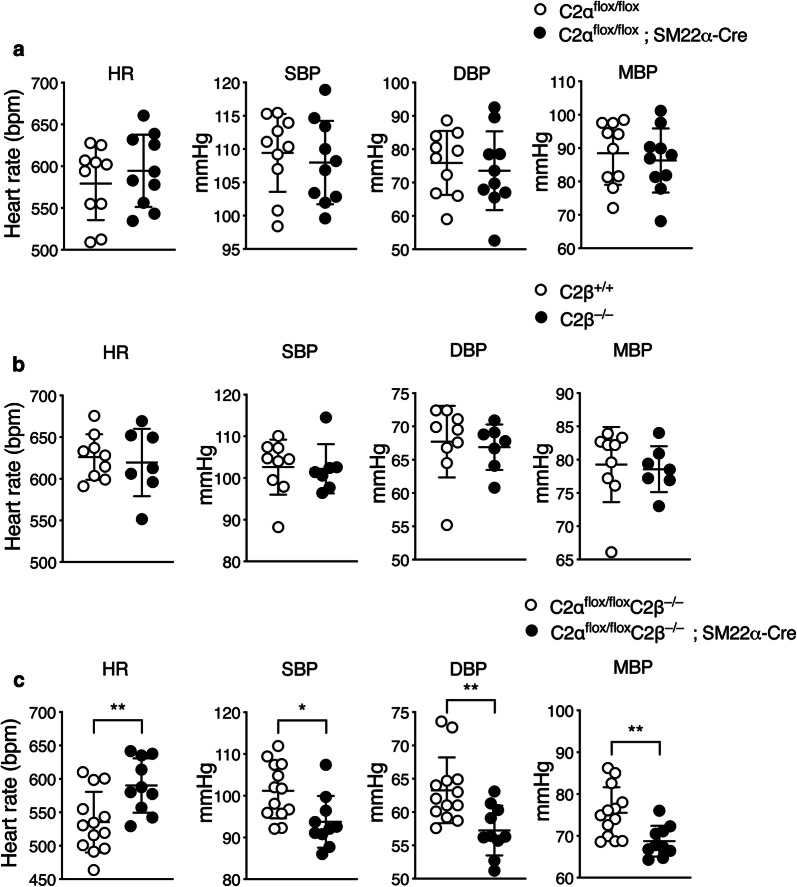
Blood pressure is decreased in DKO mice. Heart rate (HR), systolic (SBP), diastolic (DBP) and mean (MBP) blood pressure in C2α^flox/flox^ (*n* = 10) and C2α^flox/flox^; SM22α-Cre mice (*n* = 10) (**a**), in wild-type (C2β^+/+^) (*n* = 10) and C2β^−/−^ (*n* = 9) mice (**b**), and in C2α^flox/flox^ C2β^−/−^ (*n* = 13) and C2α^flox/flox^ C2β^−/−^; SM22α-Cre (*n* = 10) mice (**c**). ^★^*p* < 0.05, ^★★^*p* < 0.01

Either C2αKO mice or C2βKO mice showed no difference in systolic, diastolic or mean BP compared with control C2α^flox/flox^ and C2β^+/+^ mice, respectively (Fig. 3a, b). In contrast, DKO mice exhibited lower levels of systolic, diastolic and mean BP compared with control C2α^flox/flox^ mice (Fig. 3c).

### Attenuated contraction of aortic rings and aortic smooth muscle cells from DKO mice

**Fig. 4:**
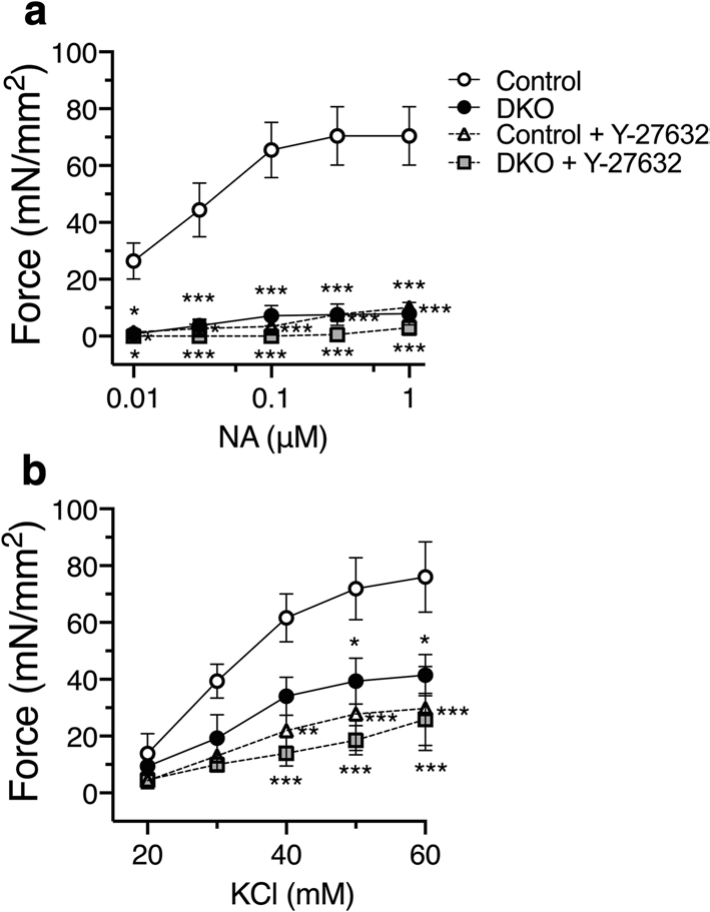
Attenuated contraction of the aortic rings from DKO mice. **a** Dose-dependent noradrenaline (NA)- and KCl **b**-induced isometric contraction of the aortic rings from C2α^flox/flox^ C2β^−/−^ (control) and DKO mice with and without the Rho kinase inhibitor Y-27632 (10 µM) was determined (*n* = 3 per each group). ^★^*p* < 0.05, ^★★^*p* < 0.01 and ^★★★^*p* < 0.001 compared with control mouse rings

We determined NA- and KCl-induced contractile responses of aortic rings isolated from DKO and control mice. NA-induced dose-dependent contraction was markedly reduced in the aortic rings from DKO mice compared with control mice (Fig. 4a). KCl-induced contraction was also reduced in DKO mice compared with control mice (Fig. 4b).

**Fig. 5:**
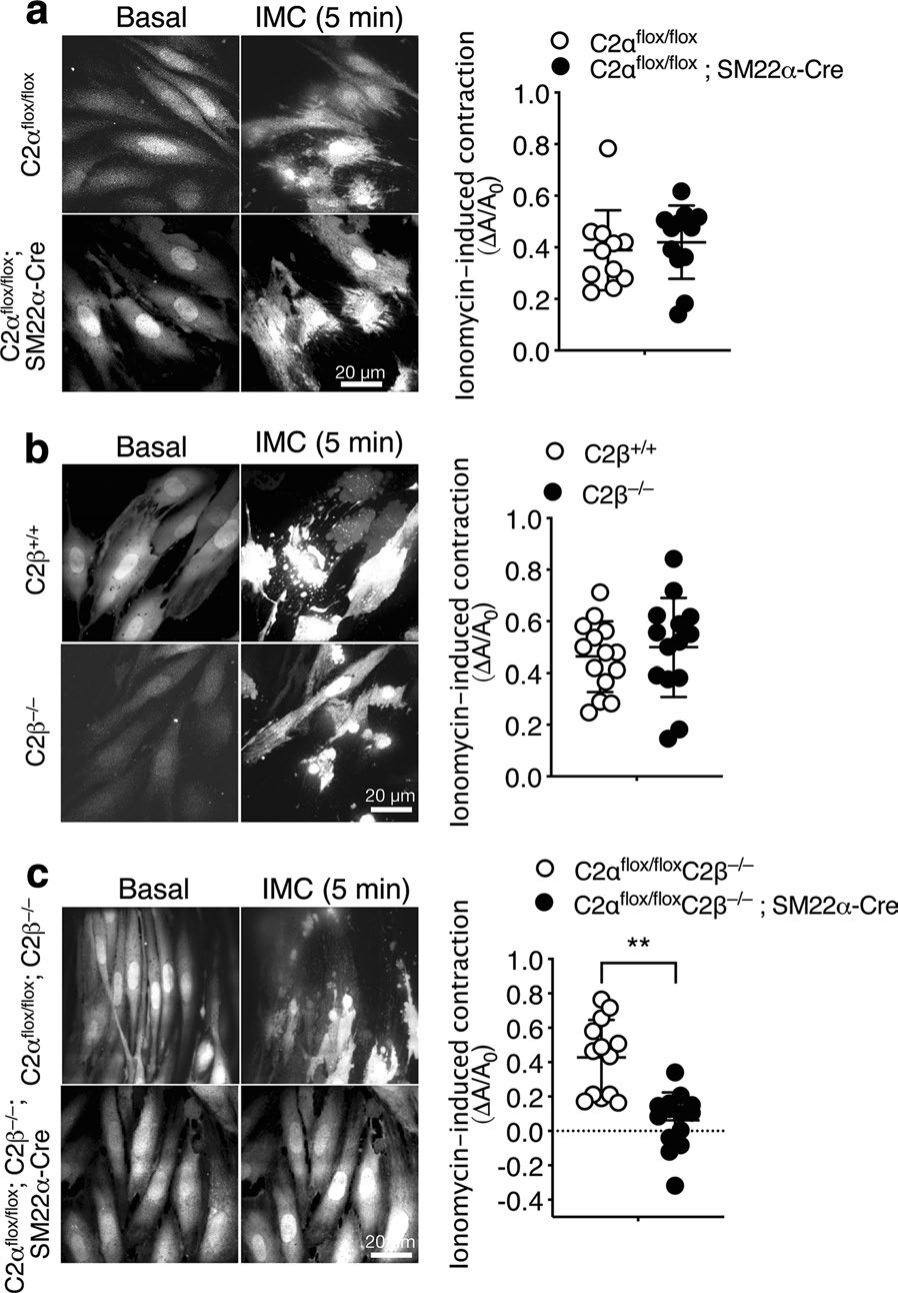
Attenuated contraction of MASM isolated from DKO mice. Fluo-8-loaded MASM were stimulated with 0.3 µM ionomycin. A difference in the planar cell surface area before and after the addition of ionomycin (∆*A*/*A*_0_) was determined in each cell and compared in C2α^flox/flox^ (*n* = 11) and C2α^flox/flox^; SM22α-Cre mice (*n* = 12) (**a**), in wild-type (C2β^+/+^) (*n* = 14) and C2β^−/−^ (*n* = 14) mice (**b**), and in C2α^flox/flox^ C2β^−/−^ (*n* = 13) and C2α^flox/flox^ C2β^−/−^; SM22α-Cre (*n* = 14) mice (**c**). Representative images of cells (left) and quantified data (right) are shown. ^★★^*p* < 0.01

We next determined contractile responses of MASM isolated from the aortae of control and KO mice. Cells were loaded with Fluo-8 to visualize cell shapes under a fluorescent microscope. The Ca^2+^ ionophore ionomycin (IMC) was employed as a stimulant because MASM lost the responsiveness to KCl-membrane depolarization and receptor agonists including NA, serotonin and a thromboxane analogue during cell culture for isolation. Ionomycin induced marked contraction of MASM from control mice with an increase in cellular Fluo-8 fluorescence (Fig. 5). To quantify cell contraction, a reduction of the planar cell surface area was determined in each cell. C2αKO cells and C2βKO cells exhibited the similar extent of IMC-induced contraction compared with each control group of cells (Fig. 5a, b). In contrast, DKO cells showed markedly reduced contraction compared with control MASMs (Fig. 5c). These findings indicate that either C2α or C2β is required for contraction, i.e., C2α and C2β are engaged in contraction of MASM in a redundant manner.

### Attenuated contraction of HASM deficient in both C2α and C2β

**Fig. 6:**
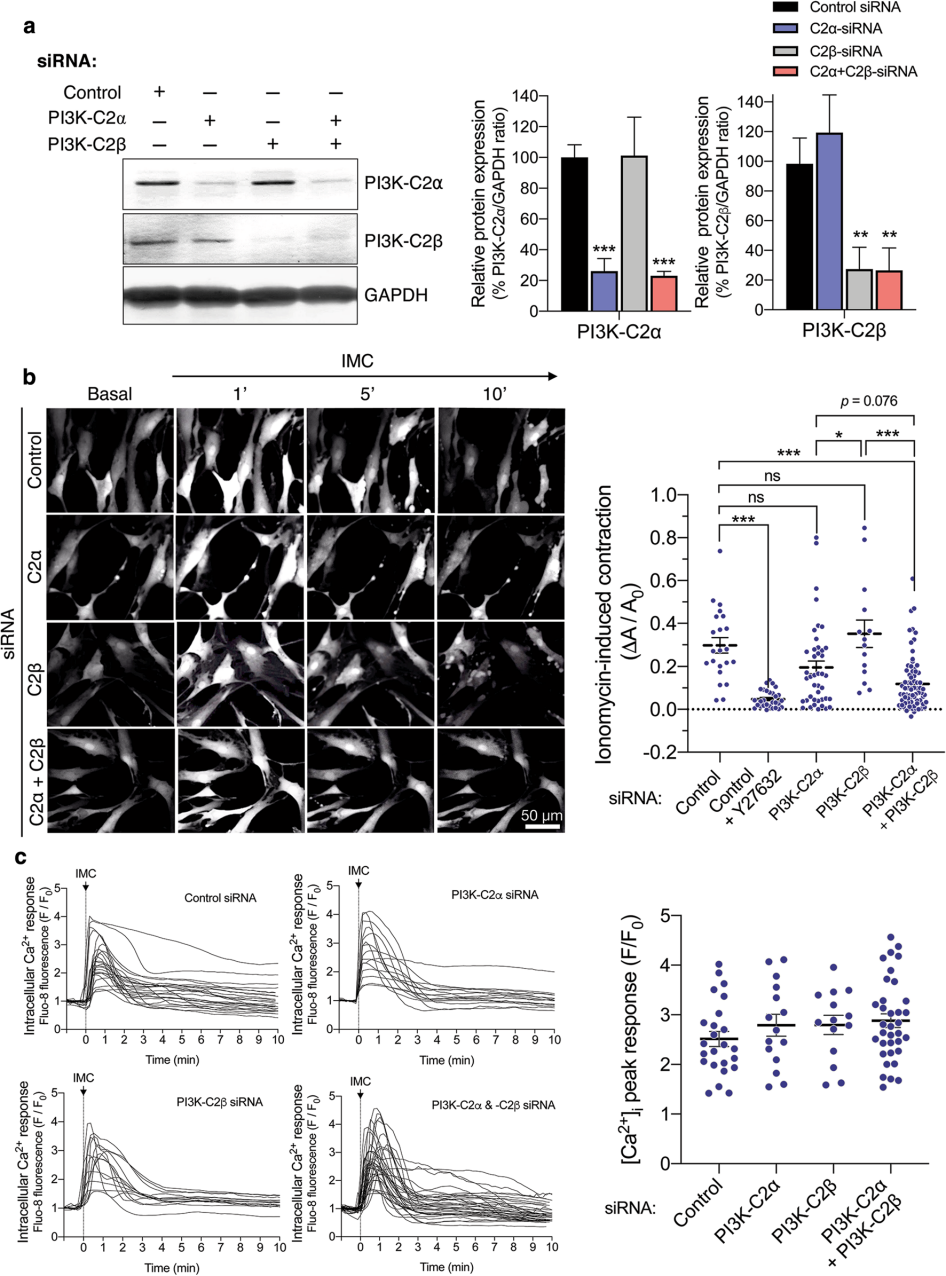
Attenuated contraction of C2α- and C2β-depleted HASM. **a** Knockdown of C2α and C2β proteins in HASM by the specific siRNAs. HASMs were transfected with control-, C2α- and C2β-siRNAs and subjected to Western blot analyses for C2α and C2β protein expression. Left, Western blotting. Middle and right panels, quantified data of C2α and C2β protein expression. **b** Attenuated contraction of C2α- and C2β-double depleted HASM. IMC (0.3 µM)-induced contraction was determined as in Fig. 5. A portion of control siRNA-transfected HASM were pretreated or unpretreated with Y-27632 (10 µM) for 5 min and stimulated with ionomycin for 10 min. Representative images of cells (left) and quantified data (right) are shown. ^★^*p* < 0.05 and ^★★★^*p* < 0.001 in the indicated comparisons. **c** No difference in ionomycin-induced increases in the [Ca^2+^]_i_ in HASM transfected with control-, C2α- and C2β-siRNAs. Left, time-dependent changes in the [Ca^2+^]_i_ in ionomycin-stimulated cells. Right, quantified data of the peak response of the [Ca^2+^]_i_

Because MASM were not suitable for further studies of C2α and C2β actions due to limited yield of cells isolated by the enzymatic dispersion method, we took advantage of HASMs which were depleted of C2α and C2β by transfection of the specific siRNAs. Both C2α- and C2β-specific siRNAs effectively and specifically depleted the expression of respective proteins by 75–80% (Fig. 6a). The combination of C2α- and C2β-specific siRNAs efficiently inhibited the expression of both C2α and C2β. Control siRNA-transfected HASM contracted robustly in response to IMC (Fig. 6b and Additional file 1: Video S1). Either singly C2α- or C2β-depleted cells showed the similar extents of contraction compared with control HASM (Additional file 2: Video S2 and Additional file 3: Video S3). In contrast, C2α- or C2β-doubly depleted HASM showed substantial attenuation of contraction compared with control and single C2α- or C2β-depleted cells (Additional file 4: Video S4). Thus, either C2α or C2β is required for full contraction of HASM similarly to the responses of mouse aortic rings and MASM and, therefore, depletion of both C2α and C2β severely impairs contraction.

### Attenuated phosphorylation of MLC_20_ and MYPT1 in HASMs deficient in both C2α and C2β

**Fig. 7:**
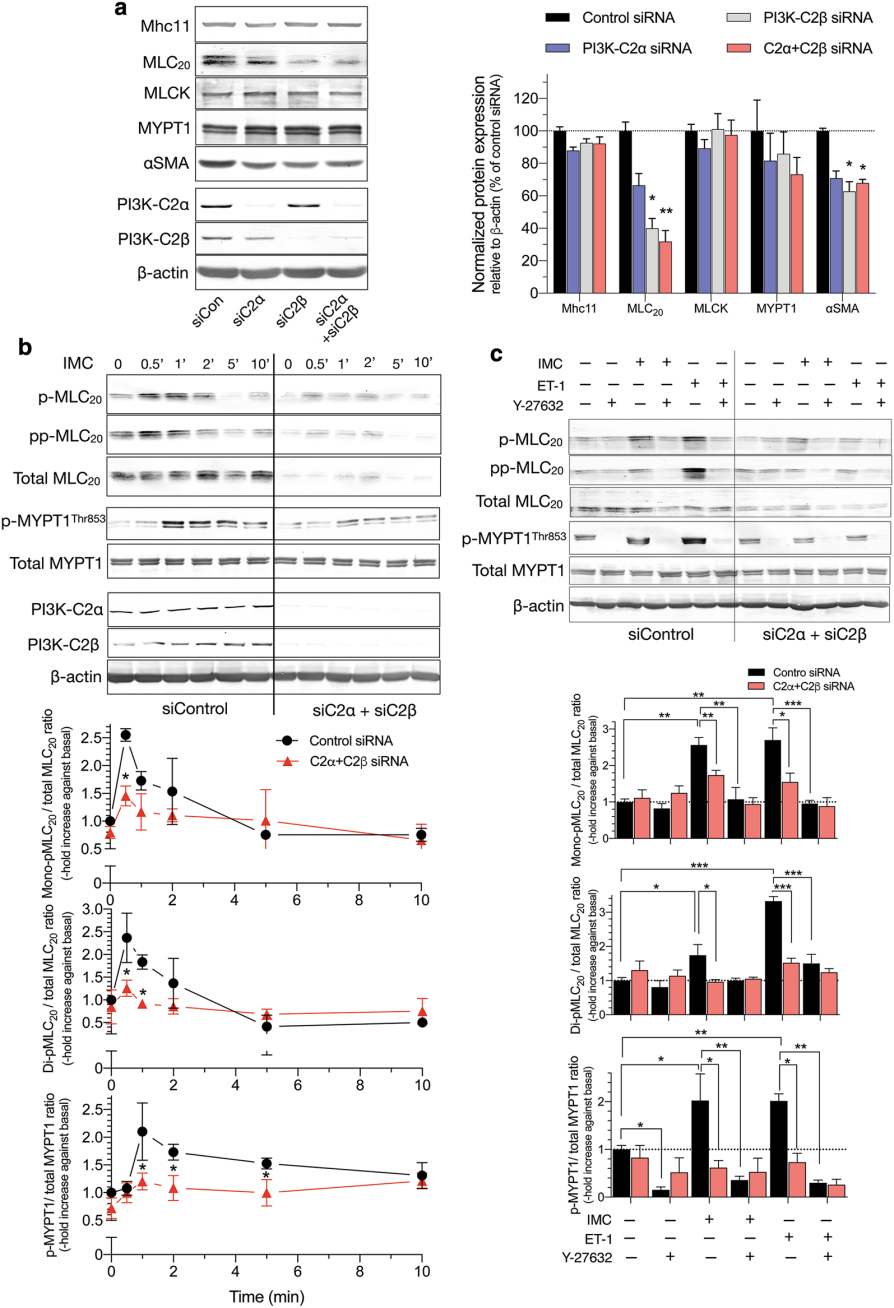
Attenuated phosphorylation of MLC_20_ and MYPT1 in C2α- and C2β-depleted HASM. **a** Effects of C2α and C2β knockdown on the expression of smooth muscle-specific proteins in HASM. Cells were transfected with control-, C2α- and C2β-siRNAs and subjected to Western blot analyses. Left, Western blots. Middle and right panels, quantified data of Mhc11, MLC_20_, MLCK, MYPT1 and αSMA. **b** Attenuated mono- and di-phosphorylation of MLC_20_ and phosphorylation of MYPT1 in C2α- and C2β-double depleted HASM. HASM were transfected with control-, C2α- and C2β-siRNAs and stimulated with 0.3 µM ionomycin for 10 min. ^★^*p* < 0.05 compared with control siRNA-transfected cells. **c** Effects of ROCK inhibitor Y-27632 on mono- and di-phosphorylation of MLC_20_ and phosphorylation of MYPT1 in C2α- and C2β-double depleted HASM. ^★^*p* < 0.05, ^★★^*p* < 0.01 and ^★★★^*p* < 0.001 in the indicated comparisons

We explored the intracellular mechanisms for the diminished contraction of C2α- and C2β-depleted HASM. Single depletion of C2α or C2β or double depletion of both C2α and C2β did not affect IMC-induced increases in the intracellular free Ca^2+^ concentration ([Ca^2+^]_i_) as determined with Fluo-8 as Ca^2+^ indicator (Fig. 6c). Depletion of C2α and C2β did not affect the protein expression of smooth muscle-specific myosin heavy chain isoform Mhc11, MLCK or MYPT1, but reduced the expression of MLC_20_ and αSMA in HASM (Fig. 7a). IMC induced time-dependent increases in mono (Ser^19^)- and di (Thr^18^, and Ser^19^)-phosphorylation of MLC_20_ in HASM (Fig. 7b). Double knockdown of C2α and C2β substantially reduced mono- and di-phosphorylation of MLC_20_ when evaluated as values corrected for total amount of MLC_20_. IMC also increased phosphorylation of MYPT1 (Fig. 7c), suggesting that IMC suppressed MLCP activity. Moreover, the Rho kinase inhibitor Y27632 substantially inhibited not only IMC-induced contraction (Fig. 6b), but also mono- and di-phosphorylation of MLC_20_ and MYPT1 phosphorylation in HASM (Fig. 7c), suggesting that Rho kinase was involved in IMC-induced MLCP inhibition, MLC_20_ phosphorylation and contraction.

### Subcellular localization of C2α and C2β in HASM

**Fig. 8:**
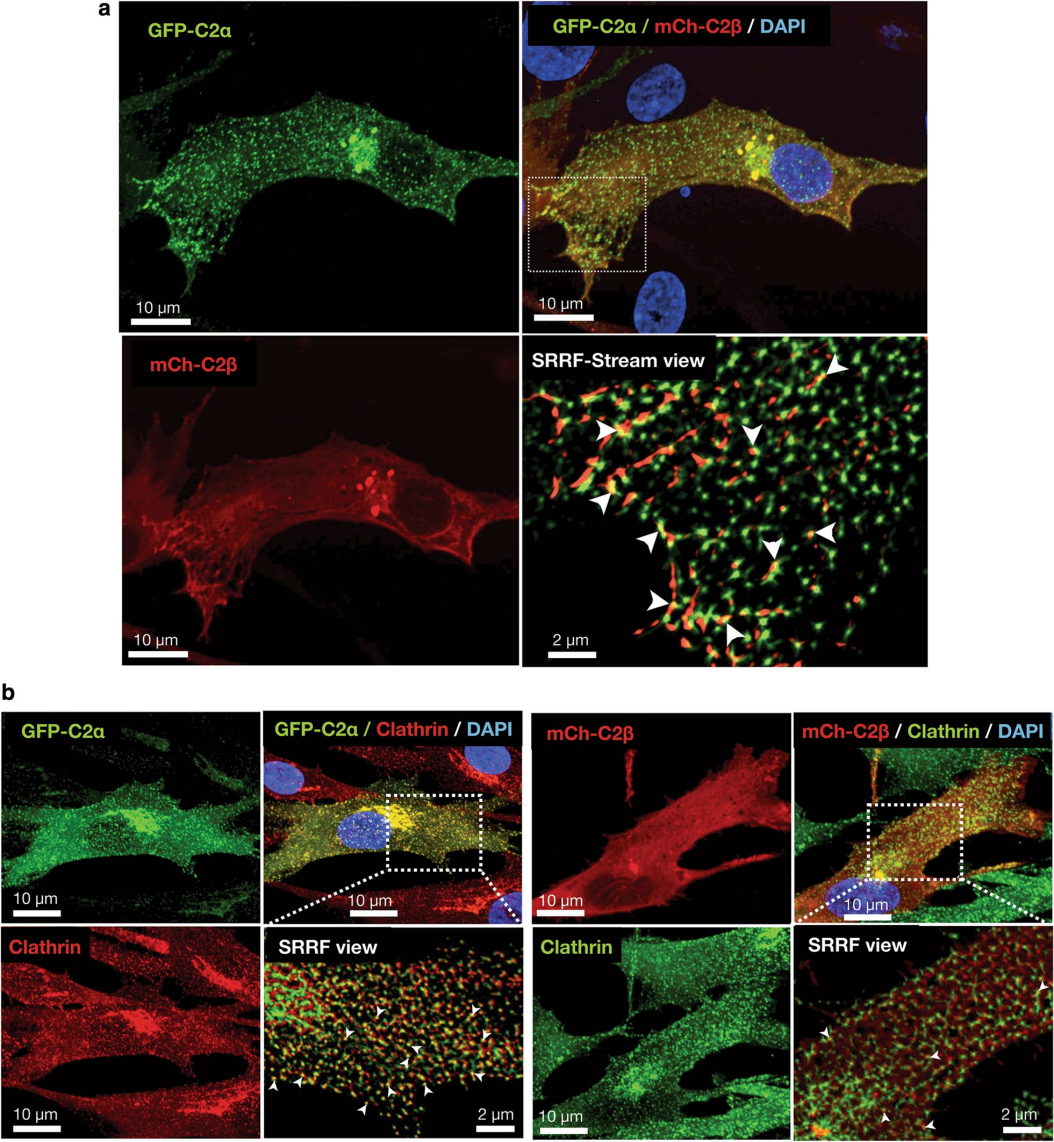
Subcellular distribution of C2α and C2β in HASM. **a** Confocal images of subcellular distribution of GFP-C2α and mCherry-C2β. Cells were co-transfected with the expression vectors for GFP-C2α and mCherry-C2β. GFP-C2α is distributed at diffuse fine puncta and perinuclear coarse puncta. mCherry-C2β (mCh-C2β) is distributed at perinuclear coarse puncta and plasma membrane. mCherry-C2β is also distributed diffusely in the mesh-like pattern with some co-localization with GFP-C2α (arrowheads in the lower right panel). **b** Co-localization of GFP-C2α and mCherry-C2β with clathrin. Cells were co-transfected with the expression vectors of GFP-C2α and mCherry-C2β, and subjected to anti-clathrin heavy chain immunofluorescence staining. Nuclei were stained with DAPI. PI3K- C2α and PI3K-C2β were co-localized with clathrin heavy chain diffusely and at the perinuclear region although the co-localization of PI3K-C2β and clathrin heavy chain was less frequent

To try to understand the role of C2α and C2β in the contractile signaling in more depth, we studied the subcellular localization of C2α and C2β in HASMs. We co-transfected HASMs with GFP-tagged C2α (GFP-C2α) and mCherry-tagged C2β (mCherry-C2β) and observed cells by confocal microscopy coupled with super-resolution radial fluctuation (SRRF-Stream) mode. We found that GFP-C2α was distributed diffusely as fine puncta and as coarse puncta locally in the perinuclear region with some signals at or near the plasma membrane in HASMs (Fig. 8a, upper left and lower views). mCherry-C2β was distributed in a fibrillary pattern in the peripheral cytoplasmic region close to the plasma membrane and in perinuclear coarse puncta with some co-localization with GFP-C2α (Fig. 8a, upper right and lower views). In the perinuclear coarse puncta and the peripheral fibrillary localization, mCherry-C2β was co-localized with GFP-C2α (Fig. 8a, upper right and lower right view). The subcellular distribution of GFP-C2α and mCherry-C2β was similar to that in human umbilical vein endothelial cells [5]. We previously observed the close co-localization of GFP-C2α and clathrin in human endothelial cells [5]. We found the similar co-localization of GFP-C2α and clathrin in HASMs (Fig. 8b). mCherry-C2β was also co-localized with clathrin, but less frequently compared with GFP-C2α. This finding was also similar to that in human endothelial cells.

### Attenuated Rho activation in HASM deficient in both C2α and C2β

**Fig. 9:**
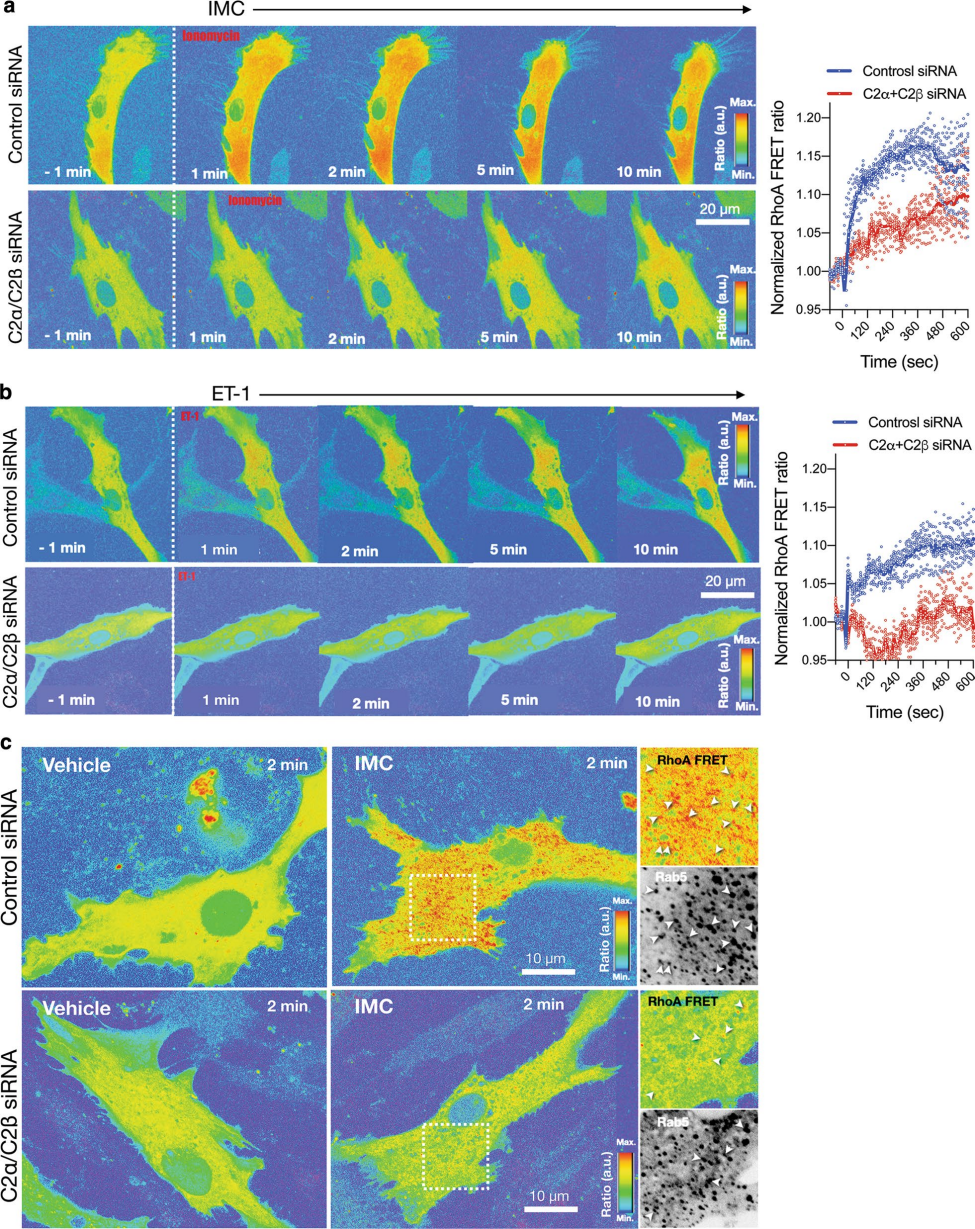
FRET imaging of attenuated Rho activation in C2α- and C2β-depleted HASM. **a** Effects of C2α and C2β knockdown on the expression of IMC (0.3µM)-induced Rho activation as evaluated with FRET imaging in control and C2α- and C2β-double depleted HASM. Left, representative images. Right, quantified data of time-dependent Rho-FRET signals. **b** Effects of C2α and C2β knockdown on ET-1 (1 µM)-induced Rho activation as evaluated with FRET imaging in control and C2α- and C2β-double depleted HASM. Left, representative images. Right, quantified data of time-dependent Rho-FRET signals. **c** Partial co-localization of RhoA-FRET signals with the early endosome marker Rab5. Cells were transfected with BFP-Rab5 expression vector and the co-localization of RhoA-FRET signals and BFP-Rab5 was determined

We determined IMC-induced Rho activation in HASMs, using a FRET imaging technique. Both IMC- and ET-1-induced Rho activation detectable within 1 min mainly in the intracellular compartment in control HASMs, and it persisted for at least 10 min of the observation time period (Fig. 9a, b, and Additional file 5: Video S5 and Additional file 6: Video S6). In contrast, in cells deficient in both C2α and C2β, IMC- and ET-1-induced Rho activation was markedly inhibited with only slight activation observed in the intracellular compartment at 5 to 10 min (Fig. 9a, b, and Additional file 5: Video S5 and Additional file 6: Video S6). In control cells, Rho-FRET signals were partially co-localized with the early endosome marker Rab5. In cells deficient in both C2α and C2β, FRET signals were greatly attenuated in both Rab5-positive and negative intracellular sites (Fig. 9c). These observations suggested that C2α and C2β were involved in Rho activation in the intracellular compartment.

## Discussion

In this study, we demonstrated that class II PI3K C2α and C2β have the essential redundant role in the blood pressure regulation and vascular smooth muscle contraction. Mice that have both smooth muscle-specific C2α deletion and global C2β deletion are not lethal and therefore allowed us to evaluate the role of C2α and C2β in blood pressure regulation. Double KO of C2α and C2β but no single KO of either gene resulted in a decrease in arterial blood pressure, indicating that at least one isoform of C2α and C2β is necessary for the maintenance of blood pressure. The diminished contractile capacity of vascular smooth muscle from DKO mice suggested that attenuated vascular smooth muscle contraction was involved in hypotension in DKO mice. Moreover, the attenuation of Rho activation and resultant Rho kinase-dependent MLCP inhibition, but not of Ca^2+^ mobilization, was suggested to underlie attenuated vascular contraction and decreased blood pressure in DKO mice. These findings, together with our recent observations of the essential redundant role of C2α and C2β in uterine smooth muscle contraction and parturition [17], point to the importance of C2α and C2β for activation of Rho and Rho kinase pathway in smooth muscle contraction in mice.

Vascular smooth muscle tone is a major determinant of the blood pressure and directly regulated by the contraction and relaxation state of vascular smooth muscle, which are under the control of various vasoconstrictors and vasodilators, sympathetic nerve activity, and mechanical stress [20–22]. The contractile responses of DKO mouse-derived aortic rings to NA and membrane depolarization were substantially reduced compared with aortic rings from control mice. Smooth muscle contraction is mediated by the two major signaling pathways of Ca^2+^-dependent MLCK activation (Ca^2+^-MLCK pathway) and Rho- and Rho kinase-dependent MLCP inhibition (Rho-MLCP pathway) [23–26]. The increase in the [Ca^2+^]_i_ activates Ca^2+^/calmodulin-dependent MLCK, leading to MLC_20_ phosphorylation, and Rho activates its effector Rho kinase, resulting in inhibition of MLCP by phosphorylating the myosin-targeting subunit MYPT1 of MLCP. Rho-MLCP pathway as well as Ca^2+^-MLCK pathway coordinately and effectively increase MLC_20_ phosphorylation, thus playing a critical role in contraction induced by various agonists including NA, endothelin-1 and thromboxane A2. We and others previously showed that an increase in [Ca^2+^]_i_ induced by membrane depolarization and a Ca^2+^ ionophore also resulted in the activation of Rho-MLCP pathway in smooth muscle [27, 28]. In C2α- and C2β-dually deficient vascular smooth muscle cells, the activity of Rho pathway but not Ca^2+^ pathway was attenuated compared with control cells, as evaluated with the FRET imaging of Rho activation and phosphorylation status of MYPT1. Therefore, it is likely that the attenuation of Rho signaling resulted in a higher MLCP activity, leading to reductions of MLC_20_ phosphorylation and contraction in C2α- and C2β-deficient vascular smooth muscle cells compared with control cells. We observed the reduced expression of MLC_20_ and αSMA in HASM with either C2β single deficiency or C2α/C2β double deficiency. It is unknown how C2β and C2α/C2β deficiency resulted in the downregulation of the smooth muscle-specific proteins and remains to be clarified. However, it is unlikely that the reduced expression of MLC_20_ and αSMA could be involved in the attenuation of contraction because single C2β depletion failed to inhibit contraction despite the reductions of MLC_20_ and αSMA expression.

We found in the present study that the Ca^2+^-ionophore ionomycin and endothelin-1-induced vigorous Rho activation mainly in the intracellular compartment of vascular smooth muscle cells. The intracellular localization of Rho activation site was consistent with our previous observations in mouse uterine smooth muscle cells [17] and human vascular endothelial cells [3], and reports by others in other types of cells [29–32]. Our study suggested that at least a part of the Rho activation sites was Rab5-positive structures, i.e., early endosomes.

The present study clearly showed that C2α and C2β were required for Rho activation in the intracellular compartment in vascular smooth muscle cells. How are C2α and C2β engaged in Rho activation at the early endosomes and other intracellular structures? Previous studies [3, 30–32] showed that C2α is preferentially localized to clathrin-coated pits and vesicles and plays an indispensable role in clathrin-dependent endocytosis. C2α mainly produces PI(3,4)P_2_ [33–37] and recruits PI(3,4)P_2_-binding domain-possessing proteins including SNX9, thus facilitating growth and maturation of clathrin-coated pits and their conversion into clathrin-coated vesicles. A functional role and intracellular localization of C2β was poorly understood compared with C2α. We recently demonstrated that C2β was localized at the focal filamentous actin (actin patches) in the peripheral cytoplasmic regions and clathrin-coated pits and vesicles in vascular endothelial cells and that C2β as well as C2α were necessary for clathrin-dependent fluid-phase endocytosis [5]. The present study showed that vascular smooth muscle cells exhibited the similar intracellular distribution of C2β to that in endothelial cells. Activation of Rho is mediated by a guanine nucleotide-exchange factor (GEF) [38]. Therefore, a GEF is likely recruited to early endosomes and other intracellular structures in stimulated vascular smooth muscle cells. In the case of ET-1-stimulated vascular smooth muscle cells, it could be possible that class II PI3K might be necessary for the endocytosis of ET-1-bound receptor into the early endosomes and that the endocytosed receptor, in turn, might recruit a GEF via the heterotrimeric G protein G_12/13_ to result in Rho activation. When vascular smooth muscle cells are stimulated with IMC, a Ca^2+^-induced Rho activation mechanism operates [12, 27]. The Ca^2+^-induced Rho activation may be mediated by a particular type of GEF and the recruitment of the particular GEF may require a process of endocytosis of the GEF molecule itself or a regulatory molecule of the GEF [39]. These endocytic processes may be dependent on PI3K-C2α and PI3K-C2β. Further studies are required for defining the exact role of C2α and C2β in the intracellular Rho activation.

Our previous studies showed that siRNA-mediated specific knockdown of only C2α-inhibited contraction of vascular smooth muscle cells derived from rat aortae [12, 13]. In contrast, the present study showed that knockdown of C2α alone was not enough to completely inhibit contraction of mouse and human vascular smooth muscle cells, which may suggest that C2β can compensate for a defect caused by C2α deficiency in mouse and human vascular smooth muscle cells; C2β may be able to compensate for insufficient PI(3,4)P_2_ production due to C2α depletion in clathrin-coated pits and vesicles and other intracellular sites in mouse and human, but not rat, vascular smooth muscle cells. It is unknown at present whether the expression levels of C2β or differences in the subcellular localization of C2β could bring about the species difference in C2α- and C2β-dependence of vascular smooth muscle contraction. The exact molecular mechanisms for the species-specific dependence on C2α and C2β of vascular smooth muscle contraction remain to be clarified.

Because the RhoA–Rho kinase–MLCP pathway is one of the major contractile mechanism in vascular smooth muscle contraction [23, 24, 26], the novel role of C2α and C2β in Rho–Rho kinase–MLCP pathway may provide some insight about understanding the pathophysiology and development of new therapies for cardiovascular diseases including hypertension and vasospasms. For example, a class II PI3K inhibitor, which inhibits Rho activation and thereby stimulates MLCP in vascular smooth muscle, may be a candidate for developing new anti-hypertensive and spasmolytic agents. Better understanding of the C2α and C2β actions at the cellular and molecular levels is required to unravel the pathophysiological role of human class II PI3K.

## Conclusions

The present study showed the importance of C2α and C2β in the regulation of the Rho–Rho kinase–MLCP pathway and contraction in vascular smooth muscle and blood pressure regulation. C2α and C2β are required for Ca^2+^- and receptor agonist-elicited Rho activation in the intracellular compartment. The role of C2α and C2β is essential for vascular smooth muscle contraction, but compensatory for deficiency of each other. Further study is required to reveal the exact mechanism of C2α- and C2β-dependent Rho activation in vascular smooth muscle.

### Supplementary information

**Additional file 1: Video S1.** The time-lapse imaging of ionomycin-induced [Ca^2+^]_i_ response and contraction of control siRNA-transfected HASM. Control siRNA-transfected, the fluorescent Ca^2+^ indicator fluo-8 loaded HASM cells were stimulated with ionomycin (0.3 µM) at 1 min, and contractile responses and changes in [Ca^2+^]_i_ response were continuously monitored for 10 min (10-s intervals).

**Additional file 2: Video S2.** The time-lapse imaging of ionomycin-induced [Ca^2+^]_i_ response and contraction of PI3K-C2α siRNA-transfected HASM. PI3K-C2α specific siRNA-transfected, the fluorescent Ca^2+^ indicator fluo-8 loaded HASM cells were stimulated with ionomycin (0.3 µM) at 1 min, and contractile responses and changes in [Ca^2+^]_i_ response were continuously monitored for 10 min (10-s intervals).

**Additional file 3: Video S3.** The time-lapse imaging of ionomycin-induced [Ca^2+^]_i_ response and contraction of PI3K-C2β siRNA-transfected HASM. PI3K-C2β specific siRNA-transfected, the fluorescent Ca^2+^ indicator fluo-8 loaded HASM cells were stimulated with ionomycin (0.3 µM) at 1 min, and contractile responses and changes in [Ca^2+^]_i_ response were continuously monitored for 10 min (10-s intervals).

**Additional file 4: Video S4.** The time-lapse imaging of ionomycin-induced [Ca^2+^]_i_ response and contraction of PI3K-C2α and -C2β siRNA-transfected HASM. PI3K-C2α and -C2β specific siRNA-transfected, the fluorescent Ca^2+^ indicator fluo-8 loaded HASM cells were stimulated with ionomycin (0.3 µM) at 1 min, and contractile responses and changes in [Ca^2+^]_i_ response were continuously monitored for 10 min (10-s intervals).

**Additional file 5: Video S5.** The time-lapse imaging of RhoA-FRET in ionomycin-stimulated HASM. HASM cells were transfected with RhoA-FRET probe expression vector and either control siRNA (left) or PI3K-C2α and -C2β specific siRNAs (right), and stimulated with ionomycin (0.3 µM) at 1 min. RhoA-FRET signals were monitored by confocal microscopy for 10 min (10 s-intervals). Note that the addition of ionomycin resulted in rapid activation of RhoA in control cells whereas ionomycin-induced RhoA activation was substantially reduced in C2α- and C2β-depleted cells.

**Additional file 6: Video S6.** The time-lapse imaging of RhoA-FRET in ET-1-stimulated HASM. HASM cells were transfected with RhoA-FRET probe expression vector and either control siRNA (left) or PI3K-C2α- and -C2β specific siRNAs (right), and stimulated with endothelin-1 (1 µM) at 1 min. RhoA-FRET signals were monitored by confocal microscopy for 10 min (10 s-intervals). Note that endothelin-1 resulted in rapid activation of RhoA in control cells whereas endothelin-1-induced RhoA activation was substantially reduced in C2α- and C2β-depleted cells.

## Data Availability

The data that support the findings of this study are available from the corresponding author on reasonable request.

## Abbreviations

PI3K: Phosphoinositide 3-kinases
C2α: Phosphoinositide 3-kinase-C2α
C2β: Phosphoinositide 3-kinase-C2β
[Ca^2^^+^]_i_: Intracellular free Ca^2^^+^ concentration
Cre: Cre recombinase
R26-tdTomato: Rosa26-CAG-loxP-stop-loxP-tdTomato
DAPI: 4′,6-Diamidino-2-phenylindole
KO: Knockout
DKO: Double knockout
FRET: Fluorescence resonance energy transfer
GEF: Guanine nucleotide exchange factor
HBSS: Hanks' balanced salt solution
MLC_20_: 20-kDa myosin light chain
MLCK: Myosin light chain kinase
MLCP: Myosin light chain phosphatase
IMC: Ionomycin
ET-1: Endothelin-1
NA: Noradrenaline
PI(3,4)P_2_: Phosphatidylinositol 3,4-bisphosphate
SRRF: Super-resolution radial fluctuation
VEGF: Vascular endothelial growth factor
GFP: Green fluorescent protein
MASM: Mouse aortic smooth muscle cells
HASM: Human aortic smooth muscle cells
αSMA: α-Smooth muscle actin
BP: Blood pressure
